# Tumor treatment by pHLIP-targeted antigen delivery

**DOI:** 10.3389/fbioe.2022.1082290

**Published:** 2023-01-06

**Authors:** Michael DuPont, Hannah Visca, Anna Moshnikova, Donald M. Engelman, Yana K. Reshetnyak, Oleg A. Andreev

**Affiliations:** ^1^ Physics Department, University of Rhode Island, Kingston, RI, United States; ^2^ Department of Molecular Biophysics and Biochemistry, Yale, New Haven, CT, United States

**Keywords:** cancer therapeutic vaccine, tumor acidity, immunization, HA peptide, alpha-gal, epitopes, immunogenic

## Abstract

Targeted antigen delivery allows activation of the immune system to kill cancer cells. Here we report the targeted delivery of various epitopes, including a peptide, a small molecule, and a sugar, to tumors by pH Low Insertion Peptides (pHLIPs), which respond to surface acidity and insert to span the membranes of metabolically activated cancer and immune cells within tumors. Epitopes linked to the extracellular ends of pH Low Insertion Peptide peptides were positioned at the surfaces of tumor cells and were recognized by corresponding anti-epitope antibodies. Special attention was devoted to the targeted delivery of the nine residue HA peptide epitope from the Flu virus hemagglutinin. The HA sequence is not present in the human genome, and immunity is readily developed during viral infection or immunization with KLH-HA supplemented with adjuvants. We tested and refined a series of double-headed HA-pHLIP agents, where two HA epitopes were linked to a single pH Low Insertion Peptide peptide *via* two Peg12 or Peg24 polymers, which enable HA epitopes to engage both antibody binding sites. HA-epitopes positioned at the surfaces of tumor cells remain exposed to the extracellular space for 24–48 h and are then internalized. Different vaccination schemes and various adjuvants, including analogs of FDA approved adjuvants, were tested in mice and resulted in a high titer of anti-HA antibodies. Anti-HA antibody binds HA-pHLIP in blood and travels as a complex leading to significant tumor targeting with no accumulation in organs and to hepatic clearance. HA-pHLIP agents induced regression of 4T1 triple negative breast tumor and B16F10 MHC-I negative melanoma tumors in immunized mice. The therapeutic efficacy potentially is limited by the drop of the level of anti-HA antibodies in the blood to background level after three injections of HA-pHLIP. We hypothesize that additional boosts would be required to keep a high titer of anti-HA antibodies to enhance efficacy. pH Low Insertion Peptide-targeted antigen therapy may provide an opportunity to treat tumors unresponsive to T cell based therapies, having a small number of neo-antigens, or deficient in MHC-I presentation at the surfaces of cancer cells either alone or in combination with other approaches.

## 1 Introduction

The targeted delivery of immunogenic epitopes to cancer cells to promote immunological responses or cytotoxic activity might provide a useful clinical approach for the treatment of tumors, and this idea has stimulated a number of investigations. Among the epitopes that have been studied are 2,4-dinitrophenyl (DNP), α-Gal (Galα1,3Galα1,4GlcNAc-R), rhamnose sugars, and other chemical entities. The α-Gal epitope has attracted special attention, since it is absent in humans, apes and Old World monkeys and it is present in non-primate mammals, prosimians and New World monkeys (Larsen et al., 1990). Humans have anti-Gal antibodies (∼1% of immunoglobulins) as immunoglobulin G (IgG), IgM and IgA isotypes (Hamanova et al., 2015). It is well known that a strong allergic reaction is developed during transplantation of organs from animals, such as pigs, to humans (xenotransplantation) due to the presence of the α-Gal epitope (Commins and Platts-Mills, 2013; Cooper, 2016), triggering complement-dependent cytotoxicity (CDC), antibody-dependent cell-mediated cytotoxicity (ADCC) and antibody-dependent cellular phagocytosis (ADCP) leading to organ rejection. Since humans exhibit specific anti-Gal reactivity, an α-Gal epitope has been developed for the decoration of cancer cells to induce an immune attack and “tumor rejection” (Macher and Galili, 2008; Tanemura et al., 2013; Huai et al., 2016; Anraku et al., 2017). Another sugar antigen that is not present in humans, L-rhamnose, is prevalent in microbes and plants, and endogenous antibodies were identified in humans against it (Oyelaran et al., 2009; Sheridan et al., 2014). Among immunogenic small molecules the most investigated is DNP, which is much easier to produce and manipulate compared to carbohydrate epitopes, especially α-Gal. However, the amount of anti-DNP endogenous IgG antibodies found in humans is smaller compared to anti-Gal antibodies (Farah, 1973; Karjalainen and Makela, 1976; Ortega et al., 1984). A challenge for therapeutic uses is to target, anchor and display immunogenic epitopes at cancer cell surfaces to induce cytotoxic immune responses within the tumor microenvironment (TME). In clinical trials with the α-Gal epitope, lipids were used to position α-Gal at cell surfaces *via* intra-tumoral administration of α-Gal-lipid (Whalen et al., 2012; Albertini et al., 2016). However, systemic applications of such an epitope-lipid composition would be limited since lipids are expected to readily insert into cellular membranes at the injection site. For systemic uses, antibody-recruiting molecules (ARMs) were devised, which are small molecules constituting of an antigen used to target receptors at the surfaces of cancer cells and DNP (or another epitope) to capture endogenous antibodies (Murelli et al., 2009; Parker et al., 2009).

A different approach for targeted epitope presentation is to use the pH-Low Insertion Peptide (pHLIP) technology. In addition to successful preclinical and clinical pHLIP-driven intracellular delivery of payloads to tumors (Cheng et al., 2014; Wyatt et al., 2018; Price et al., 2019; Sahraei et al., 2019; Gayle et al., 2021), pHLIPs allow extracellular targeting of cargo molecules for presentation at the surfaces of cells since a pHLIP peptide inserts across the membrane, translocating one terminus into the cytoplasm and leaving the other uninserted terminus in the extracellular space (Hunt et al., 1997; Reshetnyak et al., 2007; Andreev et al., 2010). It has been shown that a pH-dependent pHLIP-mediated decoration of cancer cells with DNP promotes ADCC (Wehr et al., 2020) and that a display of a chemoattractant agonist activates the formyl peptide receptor 1 on immune cells (Sikorski et al., 2022). An additional challenge for all of these immune approaches is to achieve effective presentation of the antigens. The tuning of pHLIP sequences has allowed us to improve antigen presentation to achieve enhanced therapeutic responses.

The repertoire of immunogenic epitopes might also be significantly enhanced if therapeutic efficacy did not rely on the presence of endogenous antibodies, but instead exploited the production of anti-epitope antibodies that is induced by immunization against the selected epitope. In the presented work we have compared applications of different immunogenic epitopes linked with pHLIP peptides and introduced the additional approach of using immunization to develop a strong antibody response prior to the decoration of tumor cells with an exogeneous epitope.

## 2 Materials and methods

### 2.1 Synthesis of pHLIP agents

All peptides were synthetized and purified by SC Bio Inc. Peptides pHLIP3 and pHLIP4 consisted of all D amino acids, all other peptides consisted of all L amino acids. Some peptides had an acetylated N-terminus (Ac) and some peptides had azide (Az or N_3_) modification.

#### 2.1.1 DNP-pHLIP constructs

To prepare DNP-pHLIP, DNP-maleimide (AAT Bioquest) was conjugated with a single cysteine residue at the N-terminal part of pHLIP Var3 (Cys-pHLIP) (ACDDQNPWRA YLDLLFPTDTLLLDLLWA). DNP-maleimide was mixed with Cys-pHLIP in DMSO (dimethyl sulfoxide) at a molar ratio 1:1, and sodium phosphate buffer (100 mM) at pH 6.7 containing 150 mM NaCl (saturated with argon) was added to the reaction mix (1/10 of the total volume). DNP-Peg4-NHS (NHS is N-hydroxysuccinimide) (Broadfarm) or DNP-Peg12-NHS (Santa Cruz) were conjugated with a single lysine residue at the N-terminal part of the pHLIP Var3 with acetylated N-terminus (ac-Lys-pHLIP) (Ac-AKDDQNPWRAYLDLLF PTDTLLLDLLWA) to prepare DNP-Peg4-pHLIP and DNP-Peg12-pHLIP. DNP-Peg4-NHS or DNP-Peg12-NHS were mixed with ac-Lys-pHLIP in DMSO at a molar ratio 2:1, and sodium bicarbonate buffer (100 mM) at pH 8.3 was added to the reaction mix (1/10 of the total volume).

#### 2.1.2 GAL-pHLIP constructs

To prepare di-Gal-pHLIP, di-Gal-Peg4-pHLIP or di-Gal-Peg12-pHLIP agents, first ac-Lys-pHLIP was conjugated with AMAS (N-α-maleimidoacet-oxysuccinimide ester), or NHS-Peg4-maleimide, or NHS-Peg12-maleimide cross-linkers (all from Thermo Fisher), respectively. Reactions were carried out in DMSO at a molar ratio of peptide:cross-linker of 1:3, and sodium bicarbonate buffer (100 mM) at pH 8.3 was added to the reaction mix (1/10 of the total volume). Then, di-Gal-SH (Synthose) was conjugated with AMAS-pHLIP, maleimide-Peg4-pHLIP or maleimide-Peg12-pHLIP in DMSO at a molar ratio of 1:1 in sodium phosphate buffer (100 mM) at pH 6.7 containing 150 mM NaCl buffer (1/10 of the total volume). Tri-Gal-pHLIP, where tri-Gal epitope was conjugated with pHLIP Var3 peptide *via* a Peg4 linker to obtain Gal-pHLIP (Galα(1,3)Galβ(1,4)Glc-Peg4-NHC(O)-AADDQNPWRAYLDLLFPTDTLLLDLLWA-OH), was synthesized and purified by Iris Biotech, GmbH.

#### 2.1.3 HA-pHLIP constructs

HA-pHLIP1 peptide (YPYDVPDYAGGCGGGDNDQNPWRAYLDLLFPTDTLLLDLLWA) was synthesized and purified as a single polypeptide chain. Other HA-pHLIP agents were obtained by conjugation.

Conjugation scheme N1: NHS-Peg12-maleimide and NHS-Peg24-maleimide linkers (both from Thermo Scientific) were used to prepare HA-Peg12-pHLIP2, 2(HA-Peg12)-pHLIP3, 2(3xHA-Peg12)-pHLIP3, 2(HA-Peg24)-pHLIP3, HA-Peg12-pHLIP4, 2(HA-Peg12)-pHLIP4 and 2(HA-Peg24)-pHLIP4. In brief: first, Peg-linkers were conjugated to lysine residues of acetylated pHLIP2 (Ac-AKDDQNPWRAYLDLLFPTDTLLLDL LWA), pHLIP3 (Ac-AKQNDDQNKPWRAYLDLLFPTDTL LLDLLWA) or pHLIP4 (Ac-GGKGGGKPWRAYLELLFPTETL LLELLLA) peptides in DMSO in 100 mM sodium bicarbonate buffer pH 8.3 (1/10 of total volume). Excess Peg linkers were used in the reaction mixture except for maleimide-Peg12-pHLIP3 constructs where a 1:1 ratio was used. The products were purified by reverse phase high-performance liquid chromatography (HPLC), lyophilized and conjugated with HA-Cys peptide (YPYDVPDYAGGC) or 3xHA-Cys peptide (YPYDVPDYAGYPYDVPDYAGYPYDVPDYAGGC) in DMSO in 100 mM sodium phosphate, 150 mM NaCl buffer, pH 6.7 (1/10 of total volume).

Conjugation scheme N2: DBCO-Peg12-malemide (DBCO is dibenzocyclooctyne) and DBCO-Peg24-malemide linkers (both from Iris Biotech) were used to prepare 2(HA-Peg12)-pHLIP5 and 2(HA-Peg24)-pHLIP5. First, Peg-linkers were conjugated with azide-modified lysine residues of pHLIP5 (K[N_3_]GGGGGK[N_3_]PWRAYLELLFPTETLLLELLLA) in DMSO in 100 mM sodium phosphate and 150 mM NaCl buffer, pH 8.5 (1/10 of total volume). The products were purified by the reverse phase HPLC, lyophilized and conjugated with HA-Cys peptide in DMSO in 100 mM sodium phosphate and 150 mM NaCl buffer, pH 6.7, 1/10 of total volume.

To synthesize HA-ICG-pHLIP and HA-Dy680-pHLIP, a Cys residue was added between the HA and pHLIP sequences (YPYDVPDYAGGGGGD**C**NDQNPWRAYLDLLFPTDTLLLD LLWA and YPYDVPDYAA**C**DDQNPWRAYLDLLFPTDTL LLDLLW). The peptides were conjugated with ICG-maleimide, where ICG is indocyanine green (Intrace Medical) and Dy680-maleimide, where Dy680 is IRDye680 (LI-COR) at a molar ratio 1:1 in DMSO in 100 mM sodium phosphate, 150 mM NaCl buffer, pH 6.7 (1/10 of total volume).

To synthesize ICG-2(HA-Peg12)-pHLIP3 and AF546-2(HA-Peg12)-pHLIP3, ICG (ICG-DBCO, Iris Biotech) or AF546 (AFDye546-DBCO, Click Chemistry Tools) were conjugated to the N-terminal azide of 2(HA-Peg12)-pHLIP3 peptide at a molar ratio of 1:1 in DMSO in 100 mM sodium phosphate, 150 mM NaCl buffer, pH 8.5 (1/10 of total volume).

All antigen-pHLIP constructs were purified by reverse phase HPLC, lyophilized and characterized by analytical HPLC and MALDI-TOF (matrix-assisted laser desorption/ionization-time of flight) mass-spectrometry.

### 2.2 Biophysical studies with HA-pHLIP agents

#### 2.2.1 Preparation of liposomes

Large unilamellar vesicles (liposomes) were prepared by extrusion. POPC (1-palmitoyl-2-oleoyl-*sn*-glycero-3-phosphocholine) lipids (Avanti Polar Lipids) dissolved in chloroform were desolvated on a rotary evaporator and dried under vacuum. The phospholipid film was rehydrated in 2 mM citrate phosphate buffer, pH 7.3, vortexed, and passed through the extruder using a membrane with 50 nm pores.

#### 2.2.2 Steady-state fluorescence and CD measurements

The interactions of HA-pHLIP agents with POPC liposomes were investigated by recording the construct’s spectral signals. Fluorescence spectra were recorded using a PC1 spectrofluorometer (ISS) at excitation of 295 nm. The excitation polarizer was set to 54.7 degrees (“magic angle”) while the emission polarizer was set to 0 degrees to reduce Wood’s anomalies. Circular dichroism (CD) was monitored by using a MOS-450 spectrometer (BioLogic) from 190 to 260 nm with step size of 1 nm. The concentrations of HA-pHLIP agents and POPC were 7 μM and 1.4 mM, respectively. All measurements were performed at 25°C.

#### 2.2.3 pH-dependence

The pH-dependent insertion of HA-pHLIPs into the lipid bilayer of POPC liposomes was studied by monitoring the changes of the positions of fluorescence spectral maxima (λ_*max*_) as a function of pH. After the addition of aliquots of citric acid, the pHs of solutions containing HA-pHLIP agents and POPC liposomes were measured using an Orion PerHecT ROSS Combination pH Micro Electrode and an Orion Dual Star pH and ISE Benchtop Meter. The positions of fluorescence spectra maxima were found using the PFAST program (Shen et al., 2008), and were plotted as a function of pH. The pH-dependence was fit with the Henderson-Hasselbalch equation to determine the cooperativity (*n*) and the mid-point ( pK ) of the transition:

$$pH\;dependence=SII+\frac{SIII-SII}{1+10^{n\left(pH-pK\right)}}$$

where *SII* and *SIII* represent spectral signals in state II (HA-pHLIP agents with POPC liposomes at high pH) and state III (Ha-pHLIP agents with POPC liposomes at low pH), respectively.

#### 2.2.4 Data analysis

All data was fitted to the appropriate equations by non-linear least squares curve fitting procedures employing the Levenberg Marquardt algorithm using Origin 8.5.

### 2.3 Imaging of tumor spheroids

#### 2.3.1 Preparation of tumor spheroids

Tumor spheroids were prepared using HeLa human cervical cancer cells (ATCC, CCL-2) or murine mammary carcinoma 4T1 cells (ATCC, CRL-2539) by two methods.

In the liquid overlay method, each well of a standard 48-well plate was coated by drying 150 μl of a 1% agarose solution. After drying, the agarose was incubated with growth medium containing DMEM (Dulbecco’s Modified Eagle Medium), 5% of FBS (Fetal Bovine Serum) and ciprofloxacin antibiotic for 40 min at 37°C at 5% CO_2_. The growth medium was removed and replaced by cells in DMEM (10^4^ cells in 400 μl). The plate was incubated at 37°C at 5% CO_2_ for about 5 days. After 5 days, tumor spheroids were inspected and DMEM was added to the wells. The spheroids were used for imaging experiments within approximately 10 days after seeding.

In the hanging drop method, cells in suspension were centrifuged at 200 g for 15 min to form a pellet. The supernatant was decanted, and the cells were resuspended in growth medium at concentration of 10^5^ cells per ml. A closed petri dish was prepared by adding 5 ml of PBS (phosphate buffered saline) to the bottom portion, along with inverting the top of the dish and placing it on a flat surface. The cell suspension was then pipetted onto the inverted top in evenly spaced 20 μl drops. The top of the dish was then inverted back to its original position with the drops of cell suspension hanging down. The petri dish was closed and incubated at 37°C and 5% CO_2_ for 3 days. After 3 days 10 μl of 4 × 10^4^ cells per ml were added to each drop culture. The drop cultures were incubated at 37°C and 5% CO_2_ for 10–14 days and used in imaging experiments.

#### 2.3.2 Treatment and imaging of tumor spheroids

The spheroids were placed in an Eppendorf and washed in DPBS (Dulbecco’s phosphate buffered saline), followed by incubation with non-fluorescent or fluorescent antigen-pHLIP agent at concentrations of 5 µM in PBS at pH 6.5 for 1 h. The spheroids were washed with DPBS at pH 7.4 and incubated with corresponding fluorescent anti-antigen antibody in DPBS for 1 h. The following antibodies were used with tumor spheroids: anti-DNP-Antb-550 (Al647-AntiDNP, Vector Labs), anti-Gal-Antb-647 (Al647-AntiGal, Absolute Antibody), anti-HA-Antb-550 (Dy550-AntiHA, Invitrogen) and anti-HA-Antb-650 (Dy650-AntiHA, Invitrogen). The spheroids were washed and treated for 5 min with DAPI (4′,6-diamidino-2-phenylindole, Sigma-Aldrich) in DPBS at pH 7.4, followed by final washing, plating spheroids on a 35 mm glass bottom dish and fluorescent imaging on a Nikon Ti2-E upright confocal microscope.

#### 2.3.3 Titration of tumor spheroids

The HeLa tumor spheroids were placed in an Eppendorf tube and washed in DPBS, followed by incubation with 1 µM of HA-pHLIP1 in PBS at pH 6.5 for 1 h. The spheroids were then washed with DPBS at pH 7.4 and incubated with different concentrations of fluorescent anti-HA-Antb-550 (0.035, 0.07, 0.14, 0.28, 0.56 µM). The spheroids were next washed and treated for 5 min with DAPI in DPBS at pH 7.4, followed by final washing, plating the spheroids on a 35 mm glass bottom dish and fluorescent imaging on a confocal microscope. The mean intensities per area were calculated using the ImageJ program.

### 2.4 Mouse studies

All mouse studies were conducted at the University of Rhode Island (URI) according to the animal protocol AN04-12-011approved by URI Institutional Animal Care and Use Committee (IACUC). The studies complied with the principles and procedures outlined by the National Institutes of Health for the care and use of animals. The following strains were used in the study, C57B1 and Balb/c female mice ranging in age from 7 to 9 weeks (both from Envigo RMS, Inc.), and A3galt2 knockout female and male mice.

#### 2.4.1 A3galt2 knockout breeding

A3galt2 knockout heterozygous breeding pairs were obtained from Taconic. Breeding was conducted by the Animal Care Staff at the animal care facility at URI according to the approved by IACUC animal protocol AN 1920-002. Progeny were used for experiments or to serve as breeders to maintain the colony. Mice used for breeding were in the range of 8 weeks to 8 months of age. When the progeny mice reached the age of 5–8 weeks, they were used in the experiments. Tail biopsies (less than 5 mm of the tail tip from mice 10–21 days of age) were collected for genotyping analysis, which was performed by Taconic.

### 2.5 Mouse immunization

#### 2.5.1 DNP immunization

C57Bl/6NHsd female mice were immunized (designated as day 1) by intraperitoneal (IP) injection of 200 µl of KLH-DNP (Santa Cruz), where KLH is a keyhole limpet haemocyanin, at a concentration 1 mg/ml emulsified at a ratio 1:1 with CFA (complete Freund’s adjuvant, Santa Cruz), which consists of heat-killed *Mycobacterium tuberculosis* in non-metabolizable oils. On days 14 and 21 after the first immunization, mice were boosted by IP injection of 200 µl of KLH-DNP (1 mg/ml) emulsified at a ratio 1:1 with IFA (incomplete Freund’s adjuvant, Santa Cruz).

#### 2.5.2 Gal immunization

A3galt2 knockout female and male mice were immunized by IP injection of Galα1-3Galβ-4Glc-HSA (HSA-Gal) (Dextra Laboratories) (day 1) (HSA is human serum albumin), at a concentration of 0.2 mg/ml emulsified at a ratio 1:1 with CFA. On days 14 and 21 after the first immunization, mice were boosted by IP injection of 200 μl of HSA-Gal (0.2 mg/ml) emulsified at a ratio 1:1 with IFA.

#### 2.5.3 HA immunization

The HA peptide was conjugated with KLH to obtain KLH-HA. Briefly, HA-Cys peptide was added under argon to the maleimide-activated KLH (Sigma-Aldrich) in 10 mM phosphate buffer containing 150 mM NaCl at pH 6.7 to have final concentration of 1 mg/ml of KLH-HA. The reaction mixture was kept overnight at 4°C. KLH-HA and was used with different adjuvants. Balb/c mice were immunized by IP injection of 200 μl of KLH-HA emulsified at a 1:1 ratio with CFA on day 1. Boosts were given by IP injections of 200 μl of KLH-HA emulsified at a ratio 1:1 with IFA on days 14 and 21. Separately, BALB/c mice were immunized by either intramuscular (IM) or subcutaneous (SQ) injection (two sites, 100 μl/each) of KLH-HA emulsified with AddaVax (Invivogen) at a ratio 1:1. Also, BALB/c mice were immunized by SQ injections of KLH-HA emulsified with AdjuPhos (Invivogen) at a 1:1 ratio (2 sites, 100 μl/each) or at a 2:1 ratio (2 sites, 150 μl/each). We also used commercially available and approved vaccines for canines (Vanguard CIV H3N2, Merck) and for humans (Flucelvax^®^ Quadrivalent, Seqirus). Species conversion coefficients of 12.3 and 6.8 for human and canine vaccines were used to calculate the injection doses per kg for mice, and vaccines were injected SQ in 100 μl PBS per mouse.

Blood from mice was collected prior and after final immunization. Blood samples were kept for 40 min at room temperature (RT), centrifuged at 5,000 g for 20 min at 4°C and supernatant (serum) was collected. The levels of corresponding antibodies (anti-DNP, anti-Gal and anti-HA) was measured in mouse serum by enzyme-linked immunoassay (ELISA).

### 2.6 ELISA on blood samples

#### 2.6.1 Anti-DNP ELISA

The level of anti-DNP antibodies was measured using mouse anti-DNP IgG ELISA kit (Life Diagnostic) according to the manufacturers protocol. Briefly, mouse serum samples were diluted in PBS containing 2% BSA (bovine serum albumin), the samples were treated with DNP-coated BSA-blocked 96-well plate at RT for 1 h followed washing. Next, anti-mouse IgG HRP (horseradish peroxidase) conjugate was added, and the plate was incubated at for 45 min at RT.

#### 2.6.2 Anti-gal ELISA

96-well plate (Costar) was coated with Gal-BSA (Galα1-3Galβ1-4Glc-BSA, (Dextra Laboratories) in carbonate-bicarbonate buffer at 4°C overnight, and then blocked for 2 h with blocking buffer (2% BSA in PBS). Mouse serum samples were diluted in blocking buffer containing 2% BSA and treated with Gal-coated BSA-blocked plate at RT for 2 h followed by washing. Anti-αGal human IgG1 M86 antibodies (Absolute Antibody) were used as a positive control. Next, donkey anti-mouse IgG-HRP conjugate (Southern Biotech) or goat anti-human IgG and IgM (immunoglobulin M) and IgA (immunoglobulin A) HRP conjugate (Abcam) were added for 1 h at RT.

#### 2.6.3 Anti-HA ELISA

Maleimide-activated clear strip plates (Pierce) were treated overnight at 4°C with HA peptide (YPYDVPDYAGGC) at concentration of 1 μg/ml in argon treated 100 mM phosphate buffer at pH 7.3. Then, wells were washed with PBS containing 0.2% Tween, and blocked by a 2 h treatment with 1 mg/ml of cysteine in argon treated 100 mM phosphate buffer at pH 7.3. The plate was washed, and samples diluted in PBS containing 2% BSA were applied for 2 h followed by washing and an application of secondary anti-mouse antibody at a concentration of 25 ng/ml for 1 h. Anti-HA ELISA was performed on blood serum samples collected from mice, and on single donor human plasma samples obtained from Innovative Research.

As the final steps, all ELISA plates were washed and incubated with TMB (3,3′,5,5′-tetramethylbenzidine, Invitrogen) and peroxide solution mixed at a ratio of 1:1 for up to 15 min, then stop solution (10% H_2_SO_4_) was added. The signal from the wells was quantified by absorbance measured at 450 nm using a Bio-Rad iMark microplate reader.

### 2.7 Treatment of immunized mice

#### 2.7.1 Treatment with DNP-pHLIP agents in LLC1 tumor mouse model

C57Bl/6NHsd female mice immunized with DNP-BSA and CFA/IFA adjuvants received a single subcutaneous (SQ) injection of 10^6^ murine Lewis lung carcinoma (LLC1) cells (ATCC CRL-1642) in the right flank on day 1. On day 3, mice were randomized into groups and agents including DNP-pHLIP, DNP-Peg4-pHLIP or DNP-Peg12-pHLIP (50 µM 250 µl per injection) were administered every second day by IP injections. Control mice (untreated group) did not receive any injections. All mice in the control and treated groups were euthanized at day 14, tumors were collected and weighed.

#### 2.7.2 Treatment with gal-pHLIP agents in B16F10 tumor model in knockout mice

A3galt2 knockout female and male mice immunized with HSA-Gal and CFA/IFA adjuvants received a single SQ injection of 10^6^ murine melanoma B16F10 cells (ATCC CRL-6475) in the right flank on day 1. On day 2, mice were randomized into groups and tri-Gal-pHLIP was administered *via* IP injections each day (80 μM 450 μl per injection). Control mice (untreated group) did not receive any injections. All mice in the control and treated groups were euthanized on day 12, tumors were collected and weighed.

#### 2.7.3 Treatment with HA-pHLIP agents in 4T1 tumor mouse model

BALB/c female mice immunized with KLH-HA and CFA/IFA adjuvants received a single right flank SQ injection of 10^5^ murine mammary carcinoma 4T1 cells (ATCC CRL-2539) on day 1. On day 3 or 4, the mice were randomized into groups and HA-pHLIP agents (40–60 µM 400 μl per injection) were administered every second day by IP injection. The control (untreated group) did not receive any injections. In additional control experiments we investigated whether selected HA-pHLIP agents affect tumor growth in non-immunized mice, and whether multiple-injections of HA-pHLIP could induce the production of anti-HA antibodies. In each experiment, the mice from the control and treated groups were euthanized on the same day (typically days 17–19), tumors were collected and weighed. All data were normalized to the mean weight of the tumors in the control group.

#### 2.7.4 Treatment with HA-pHLIP agents in B16F10 melanoma tumor mouse model

BALB/c female mice immunized with KLH-HA and the AddaVax adjuvant received a single right flank SQ injection of 4 × 10^5^ murine melanoma B16F10 cells. Tumors were grown until they reached about 100 mm^3^ (day 1). On day 1, the mice were randomized into groups and injections of the 2(HA-Peg12)-pHLIP5 agent (40 μM 400 μl per injection) were administered on three consecutive days (days 1, 2 and 3). The control (untreated group) did not receive any injections. In each experiment, the mice from the control and treated groups were euthanized on day 7, tumors were collected and weighed. All data were normalized to the mean weight of tumors in the control group.

#### 2.7.5 Statistical analysis

The Kolmogorov-Smirnov two-tailed non-parametric test was used to compare two samples at a significance level of 0.05. As a non-parametric test, it does not require that the variable in the population have a normal distribution, which is typically difficult to achieve in small population samples.

### 2.8 *In vivo* and *ex vivo* imaging, biodistribution

Tumors were established by SQ injection of 10^5^ 4T1 cancer cells in the right flanks of BALB/c mice. When tumors reached about 200–250 mm^3^, mice were given a single IP injection of 50 µM DNP-Peg12-pHLIP mixed with anti-DNP-Antb-ICG in 400 µl of PBS or the same amount of anti-DNP-Antb-ICG alone in PBS. The anti-DNP antibody was labeled with ICG-NHS (Intrace Medical) and purified using Sephadex G-50 size-exclusion spin column. Imaging was carried out at 24- and 48-h post-injection (p.i.). White light and near infrared (NIR) *in vivo* images were obtained while the animal was under gas (isoflurane) anesthesia. Mice were euthanized 48 h post injection, tumors were resected and *ex vivo* imaging was performed immediately after necropsy.

Tumors were established by SQ injection of 10^5^ 4T1 cancer cells in the right flanks of BALB/c non-immunized mice and mice immunized with KLH-HA/CFA and KLH-HA/IFA or KLH-HA/AddaVax. HA-ICG-pHLIP (80 μM 400 μl) or ICG-2(HA-Peg12)-pHLIP4 (40 μM 400 μl) were admnisitered as single IP injections when tumors reached 200–300 mm^3^ in volume. Animals were euthanized at 24-, 48- and 72-h p.i., necropsy was performed immediately after euthanasia. Tumors and major organs (kidney, liver, spleen, pancreas, lung, heart, large and small intestines, bone, muscle, brain) were collected and imaged *ex vivo* immediately after necropsy.

Bright field and NIR fluorescence imaging *in vivo* and *ex vivo* were performed using a Stryker 1588 AIM endoscopic system with L10 AIM Light Source (808 nm excitation and light collection in the range of approximately 815–850 nm), and a 1588 AIM Camera using a 10 mm scope. The lens was kept at a fixed distance from the surface of the organ, within an enclosed (light-protected) area. The imaging was performed at three different settings. Digital images of organs were saved in the green channel, transferred into 8-bit files and processed using the ImageJ program. A threshold was set from pixel intensity in the range from 1 to 255, leaving out the background with pixel intensity 0. Brightfield images were used to establish the borders of the organs and tumors. The calculated total fluorescence intensity and total area of each organ were used to find the mean organ fluorescence.

### 2.9 Immuno-histochemistry and imaging of tumor sections

For immunohistochemistry analysis, 10^5^ 4T1 cancer cells were injected SQ into the right flank of female BALB/c mice. When the tumors reached 200–300 mm^3^ in volume, mice received a single IP injection of AF546-2(HA-Peg12)-pHLIP4 (40 μM 400 μl). Animals were euthanized at 24-, 48- and 72-h p.i. and tumors were collected. Tumors were frozen in optimal cutting temperature (OCT) compound (Tissue-Tek) by liquid nitrogen and kept at −80°C until further processing and analysis. Tumors were cryo-sectioned using a HM525 NX microtome (ThermoFisher) to make 10–20 μm sections. Sections were stained with fluorescent antibodies anti-HA-Antb-650 (Dy650-AntiHA, Invitrogen) or CD206-Antb-647 (Al647-CD206, BioLegend) and DAPI. Adjacent sections were stained with H&E (hematoxylin and eosin) using hematoxylin 7211 (ThermoFisher) and eosin Y (Poly Scientific). Sections were dried in air for 10 min, then washed with distilled water for 2 min followed by fixation in 4% paraformaldehyde (37% Sigma-Aldrich) for 12 min, washing with DPBS for 5 min and drying in air for 10 min. A cover slide was placed on a layer of petroleum jelly (Equate), which was applied to the slide around the tissue. Sections were incubated with blocking buffer containing 5% of BSA (Thermo Fisher) for 2 h at RT followed by washing. Sections were treated with antibody in blocking buffer for 2 h at RT, followed by washing. A coverslip was mounted on top of the tissue using an organo/limonene mount (Sigma). Imaging of the tissue sections was performed on an EVOS Fl Auto 2 fluorescence inverted microscope. Slides used for Trypan Blue assay were not stained, mounted or covered, these slides were left frozen and imaged before and after addition of Trypan Blue diluted in PBS at a ratio of 1:5 for 5 min.

## 3 Results

### 3.1 DNP-pHLIP

**FIGURE 1:**
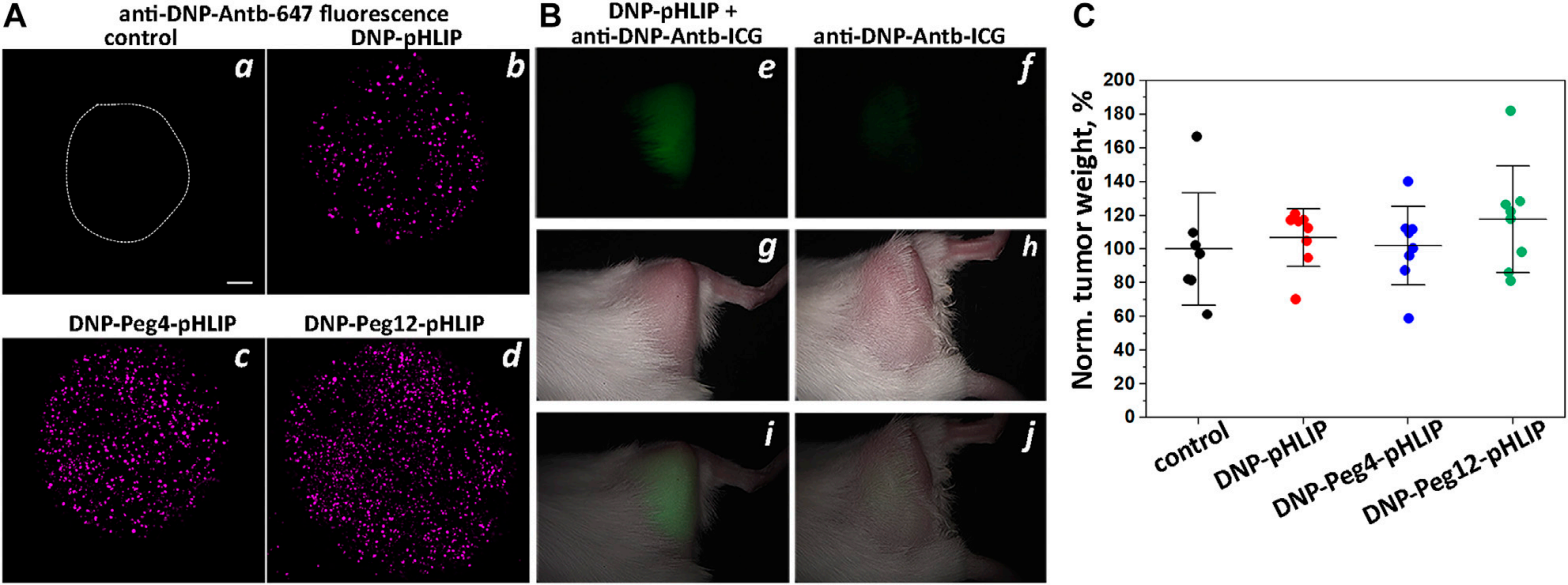
DNP-pHLIP in tumor spheroids and mice. Antibody binding and therapeutic performance of DNP-pHLIP agents as investigated in tumor spheroids **(A)** and in mice **(B,C)**. **(A)** HeLa tumor spheroids were treated with medium (the contour of the spheroid is outlined) (a), DNP-pHLIP (b), DNP-Peg4-pHLIP (c) and DNP-Peg12-pHLIP (d), followed by washing and incubation with anti-DNP-Antb-647. Finally, tumor spheroids were washed and imaged with a confocal microscope using a ×10 objective. A 100 μm scale bar is shown. Representative fluorescence images are shown in (a–d). **(B)** Representative *in vivo* NIR fluorescence (e,i), visible light photograph (g,h) and overlay of fluorescence and photograph (i,j) images obtained 24-h after a single IP injection of anti-DNP-Antb-ICG mixed with DNP-Peg12-pHLIP (e,g,i) or anti-DNP-Antb-ICG (f,h,j) into Balb/C female mice bearing 4T1 tumor in right (shaved) flank. **(C)** Normalized weights of murine Lewis lung carcinoma LLC1 tumors collected from C57Bl/6NHsd female mice immunized with KLH-DNP/CFA and KLH-DNP/IFA and treated with DNP-pHLIP, DNP-Peg4-pHLIP and DNP-Peg12-pHLIP are shown. The mean weight of the tumors in the control group was taken as 100%.

In our exploration of the use of pHLIP-targeted epitopes, we tested DNP, Gal and HA peptide. Taking them in that order, we begin with the DNP epitope linked to the membrane non-inserting end of the pHLIP Var3 peptide, either directly conjugated or spaced by Peg4 and Peg12 linkers. The epitopes were tethered to cancer cells in tumor spheroids by pHLIP and the interactions of the DNP epitope with anti-DNP antibody were examined by fluorescence microscopy. Tumor spheroids were pre-treated with DNP-pHLIP followed by washing and incubation with anti-DNP antibody labeled with fluorescent Alexa647 dye (anti-DNP-Antb-647). The fluorescence was imaged with a confocal microscope after washing. Each of the constructs, DNP-pHLIP, DNP-Peg4-pHLIP and DNP-Peg12-pHLIP, bound the anti-DNP antibody to cells in the spheroids (Figure 1A).

Imaging of tumors in mice was performed with DNP-Peg12-pHLIP, which was pre-mixed with the anti-DNP antibody labeled with the NIR fluorescent ICG dye (anti-DNP-Antb-ICG) and given as a single IP injection into mice bearing 4T1 breast tumors in their right flanks. Anti-DNP-Antb-ICG was used as a control. The data indicates tumor targeting by DNP-Peg12-pHLIP pre-mixed with anti-DNP-Antb-ICG (Figures 1B, e, g, i), while a much lower signal was seen in the control animals (Figures 1B, f, h, j). Whole body NIR fluorescence imaging revealed tumor targeting as shown in Figure 1B and confirmed by observation of the resected tumors (data not shown). Pre-mixing was thought to be important since most epitope-pHLIP molecules are expected to bind corresponding anti-epitope antibody in blood before tumor targeting.

Despite effective binding of antibody to DNP-pHLIP agents inserted into cell membranes *in vitro*, despite the previously demonstrated ADCC induced by DNP-pHLIP on cells (Wehr et al., 2020), and despite the targeted delivery of the antibody-DNP-pHLIP complex to the tumors, no therapeutic effect was observed in mice immunized against the DNP epitope (Figure 1C). Immunization was performed by several injections of KLH-DNP co-injected with CFA (first injection) and then co-injected with IFA in a series of boosts. A high blood titer of anti-DNP IgG antibodies was confirmed by ELISA performed against the DNP epitope. Mouse Lewis lung carcinoma LLC1 tumor cells were implanted and treatment with DNP-pHLIP, DNP-Peg4-pHLIP and DNP-Peg12-pHLIP was administered on day 3, constituting of IP injections of agents (50 μM 250 μl per injection) every second day for 2 weeks. On day 16, the mice were euthanized, and the tumor was collected and weighed. Figure 1C presents normalized weights of tumors in all groups. The mean tumor size in the control (untreated) group was taken as 100%. The results clearly indicate a lack of therapeutic efficacy of DNP-pHLIP agents. Such a negative therapeutic outcome might be attributed to the binding of a small hydrophobic molecule such as DNP (and DNP-pHLIP agents) to proteins in the blood and/or partitioning into cellular membranes, limiting the amount of free and antibody-bound fractions of DNP-pHLIP, and as a result, limiting the delivery of sufficient DNP epitope to tumors to induce therapeutic responses. Issues with the use of DNP as an epitope were previously reported as well (Jung et al., 2008; Zhang et al., 2010).

### 3.2 Gal-pHLIP

**FIGURE 2:**
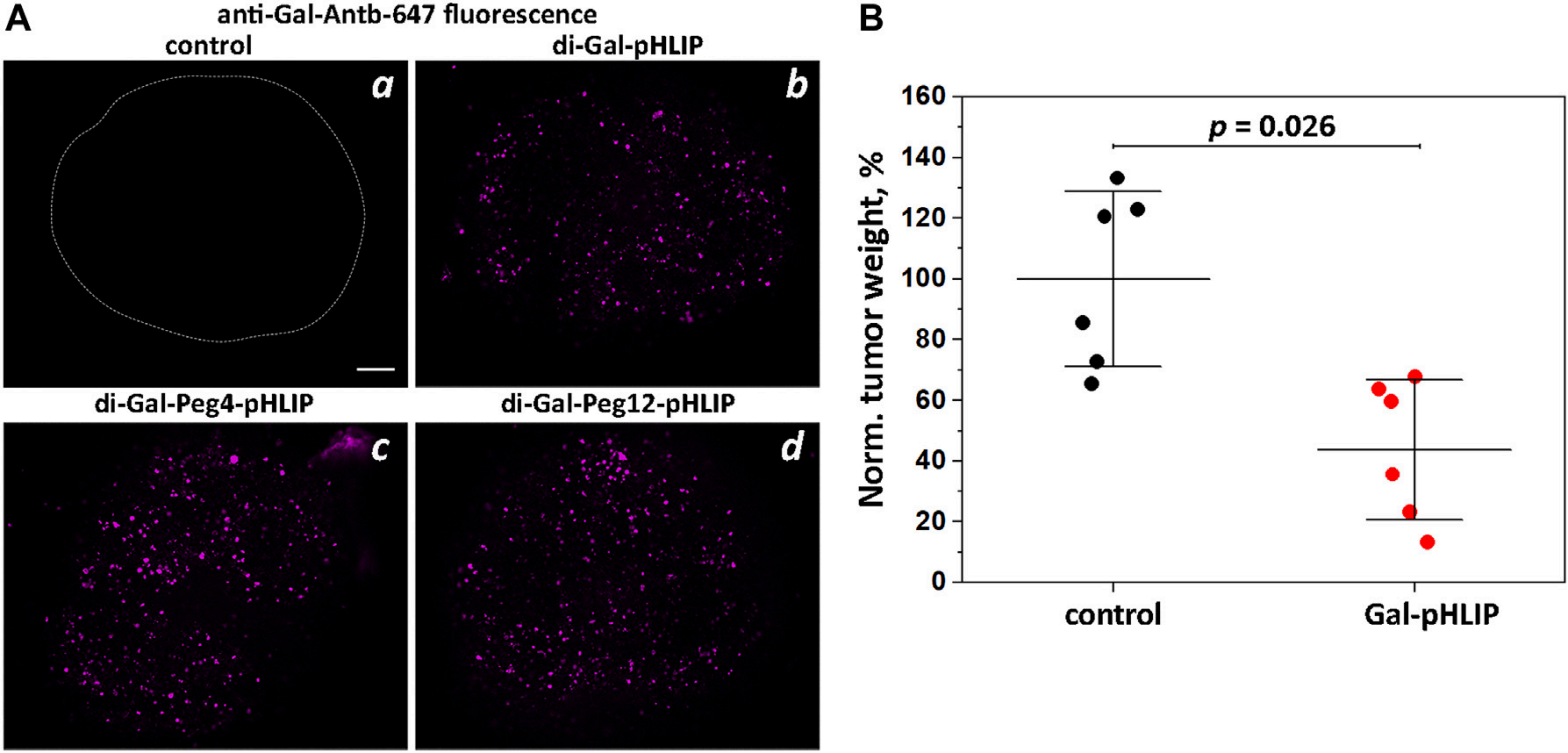
Gal-pHLIP in tumor spheroids and mice. Antibody binding and therapeutic performance of Gal-pHLIP agents investigated in tumor spheroids **(A)** and in mice **(B)**. **(A)** HeLa tumor spheroids were treated with medium (a), di-Gal-pHLIP (the contour of the tumor spheroid is outlined) (b), di-Gal-Peg4-pHLIP (c) and di-Gal-Peg12-pHLIP (d), followed by washing and incubation with anti-Gal-Antb-Al647. Finally, tumor spheroids were washed and imaged using a confocal microscope with a ×10 objective. A 100 μm scale bar is shown. Representative fluorescence images are shown in (a–d). **(B)** Normalized weights of murine melanoma B16F10 tumors collected from A3galt2 knockout mice immunized with HSA-Gal/CFA and HSA-Gal/IFA and treated with Gal-pHLIP are shown. The mean weight of tumors in the control group was taken as 100%. The distributions of tumor weights in the control (non-treated) group and Gal-pHLIP (treated) group are statistically different as established by the Kolmogorov-Smirnov two-tailed non-parametric test.

The next logical choice was the polar α-Gal epitope, which was expected to have fewer potential binding or partitioning problems than DNP. α-Gal disaccharide epitopes were conjugated to the amino terminal end of the pHLIP Var3 peptide either directly or as extended by Peg4 and Peg12 linkers. Tumor spheroids were treated with the di-Gal-pHLIP agents followed by washing, incubation with fluorescent anti-Gal-Antb-647, and final washing, and were then imaged using a confocal fluorescence microscope. Each of the agents, di-Gal-pHLIP, di-Gal-Peg4-pHLIP and di-Gal-Peg12-pHLIP, bound anti-Gal antibody when they were positioned at the surfaces of cancer cells within tumor spheroids (Figure 2A).

In order to assess therapeutic efficacy, we used an α-Gal trisaccharide epitope, known to enhance the binding affinity of anti-Gal antibodies (Anraku et al., 2017). The tri-Gal epitope was conjugated with pHLIP peptide *via* a Peg4 linker to obtain Gal-pHLIP. The Gal-pHLIP therapeutic efficacy was tested in immunized A3galt2 knockout mice using a B16F10 melanoma tumor model. Only knockout mice can produce anti-Gal antibodies, since they lack α-Gal epitopes (Thall et al., 1995; Porubsky et al., 2007). The poorly immunogenic B16 or B16F10 melanoma cells are suitable for testing Gal-pHLIP, since these cells lack α-Gal epitopes (Gorelik et al., 1995). Immunization was performed by several injections of HSA-Gal co-injected with CFA (first injection) and HSA-Gal co-injected with IFA in a series of boosts. A high blood titer of anti-Gal IgG antibodies was confirmed by ELISA against the α-Gal epitope. Murine melanoma B16F10 tumor cells were implanted into mice and the treatment with Gal-pHLIP was initiated on the next day. The treatment consisted of IP injections of the agent (80 μM 450 μl per injection) each day for 10 days. Mice were euthanized on day 12, and tumors were collected and weighed. Figure 2B presents the normalized tumor weights. The mean tumor size in the control (untreated) group was taken as 100%. The treatment led to a statistically significant reduction of tumor growth, by 66%.

### 3.3 HA-pHLIP

**FIGURE 3:**
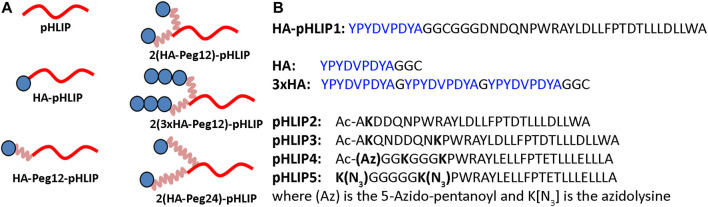
HA-pHLIP constructs **(A)**: Schematic representation of the HA-pHLIP agents, where HA is shown as blue circle, Peg linkers are shown in pink and pHLIP is shown in red. **(B)**: Sequences of HA-pHLIP synthesized as a single peptide, the separately conjugated HA and 3xHA units, and several pHLIP peptides are shown. As indicated, some pHLIP peptides had acetylated N-termini (Ac). Peptides pHLIP4 and pHLIP5 consisted of all D amino acids and the other peptides consisted of all L amino acids.

The repertoire of therapeutic immunogenic epitopes could be significantly expanded if efficacy did not rely on the presence of natural antibodies but was instead based on anti-epitope endogenous antibodies induced by immunization against the selected epitope. We have selected an exogenous antigen: a 9 amino acid HA peptide (YPYDVPDYA) from the hemagglutinin protein of H3N2 influenza virus. The HA sequence is not present in the human genome, and immunity is readily developed during viral infection or immunization against the HA antigen. We designed and tested several HA-pHLIP agents (Figure 3). HA constructs were designed to explore several ideas of how efficacy might be produced and enhanced. As a simple construct, a single HA epitope was synthesized as a continuation of the pHLIP sequence separated by a poly-Gly motif (HA-pHLIP1). In other sets of constructs, HA epitope was separated from pHLIP by Peg12 polymer (HA-Peg12-pHLIP). In an effort to enhance antibody binding to HA, double HA epitopes were linked to pHLIP (pHLIP3 or pHLIP4) *via* Peg12 linkers (2(HA-Peg12)-pHLIP) or Peg24 linkers (2(HA-Peg24)-pHLIP). Also, HA epitopes repeated three times were tried (3xHA) and conjugated with pHLIP (pHLIP3) *via* a Peg12 linker (2(3xHA-Peg12)-pHLIP. The double headed HA pHLIP peptides were designed to enhance overall antibody affinity by binding to both antigen sites of an anti-HA antibody. We used links of different lengths, Peg12 (∼5.3 nm) and Peg24 (∼9.5 nm), for conjugation of HA epitopes to pHLIPs (the distance between the antibody binding sites is about 10–11 nm). Based on biophysical measurements, we modified pHLIP sequences to achieve physiologically relevant *pK*s by enhancement of the hydrophobicity of pHLIP sequences linked to polar HA-Peg conjugates. The hydrophobicity of pHLIP4 was increased by replacing Asp residues by Glu residues, omitting a C-terminal Trp residue, and removing polar, charged residues from the N-terminal sequence. pHLIP5 is a slightly modified version of pHLIP4 with a longer poly-Gly motif between the Lys residues where HA-Peg linkers are attached to reduce steric hindrance and improve conjugation. HA-pHLIP and pHLIP4 peptides were also used for conjugation with fluorescent dyes, AFDye546 (AF546), Dy680 or ICG. Several different synthetic schemes, as described in Methods, were utilized for conjugation of HA *via* Peg linkers to Lys or azide groups at different pHLIP peptides to obtain the different agents summarized in Table 1 and Supplementary Figure S1.

**TABLE 1:** List of all HA-pHLIP constructs used in this study. The conjugated molecules are shown in bold. Az is the 5-Azido-pentanoyl and K[N_3_] is azidolysine. The calculated molecular weight (MW_c_) and molecular weight measured by masspec (MW_m_), retention time (R_t_) and purity (%) established by analytical HPLC are shown.

HA-pHLIP construct	Sequence	Peg linker	MW^a^	MW^2^	R_t_	Purity
HA-pHLIP1	YPYDVPDYAGGGGGDNDQNPWRAYLDLLFPTDTLLLDLLWA	—	4602	4602	16.3	98.4
HA-Peg12-pHLIP2	AK[HA-Peg12]DDQNPWRAYLDLLFPTDTLLLDLLWA	Maleimide-Peg12-NHS	5430	5432	16.2	99.8
2(HA-Peg12)-pHLIP3	AK[HA-Peg12]QNDDQNK[HA-Peg12]PWRAYLDLLFPTDTLLLDLLWA	Maleimide-Peg12-NHS	7871	7877	14.6	99.9
2(3xHA-Peg12)-pHLIP3^1^	AK[3xHA-Peg12]QNDDQNK[3xHA-Peg12]PWRAYLDLLFPTDTLLLDLLWA	Maleimide-Peg12-NHS	12435	—	14.4	99.9
2(HA-Peg24)-pHLIP3	AK[HA-Peg24]QNDDQNK[HA-Peg24]PWRAYLDLLFPTDTLLLDLLWA	Maleimide-Peg24-NHS	8926	8939	14.4	99.9
HA-Peg12-pHLIP4, v1	(Az)GGK[HA-Peg12]GGGKPWRAYLELLFPTETLLLELLLA	Maleimide-Peg12-NHS	5409	5406	16.2	99.2
HA-Peg12-pHLIP4, v2	(Az)GGKGGGK[HA-Peg12]PWRAYLELLFPTETLLLELLLA	Maleimide-Peg12-NHS	5409	5408	16.4	92.4
2(HA-Peg12)-pHLIP4	(Az)GGK[HA-Peg12]GGGK[HA-Peg12]PWRAYLELLFPTETLLLELLLA	Maleimide-Peg12-NHS	7480	7472	15.9	99.8
2(HA-Peg24)-pHLIP4	(Az)GGK[HA-Peg24]GGGK[HA-Peg24]PWRAYLELLFPTETLLLELLLA	Maleimide-Peg24-NHS	8537	8524	15.8	99.1
2(HA-Peg12)-pHLIP5	K(N_3_)[HA-Peg12]GGGGGK(N_3_)[HA-Peg12]PWRAYLELLFPTETLLLELLLA	Malemide-Peg12-DBCO	7902	7902	16.1	99.6

^a^ The agent did not provide good signal on masspec.

**FIGURE 4:**
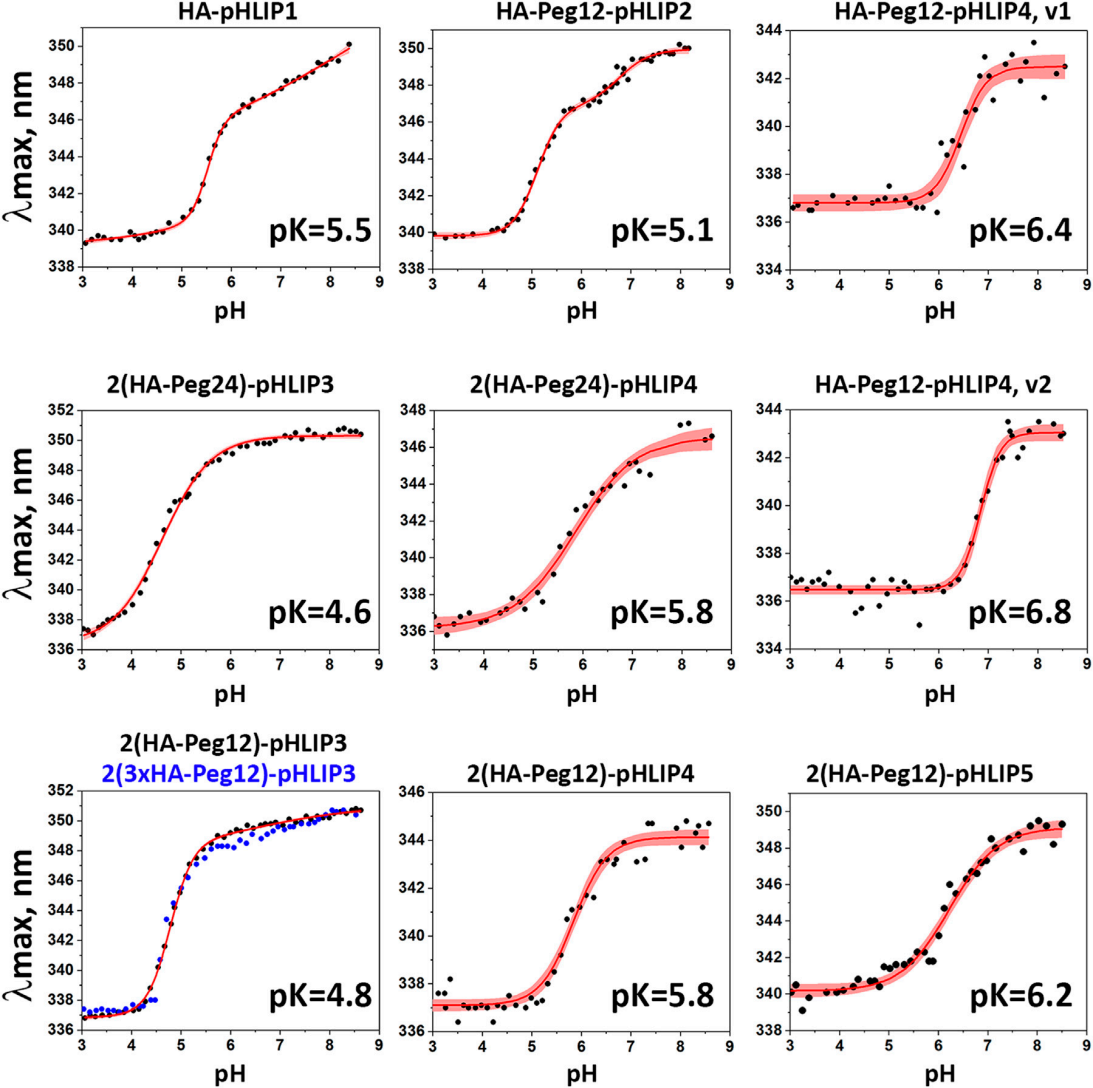
pH-dependent interactions of HA-pHLIP constructs with liposome bilayers. pH transitions from state II to state III as monitored by changes of the fluorescence maximum of HA-pHLIP agents are shown (experimental points and fitting curves, red, with 95% confidence interval, pink).

Since all pHLIP peptides contain one or two tryptophan residues, we used tryptophan fluorescence to monitor the interactions of different HA-pHLIP constructs with lipid bilayers in POPC liposomes. All agents exhibited pH-dependent pHLIP-like properties. The maximum positions of fluorescence of HA-pHLIP agents in state I (where the agent is a largely unstructured polymer in aqueous solution at pH8), state II (where the agent is adsorbed by POPC liposomes at pH8), and state III (where the agent is inserted across the lipid bilayer of POPC liposomes at pH3) are given in Table 2. Transitions from the membrane-adsorbed (state II) to the membrane-inserted (state III) conformations of HA-pHLIP agents were monitored by the shift of the maximum position of fluorescence spectra (Figure 4; Table 2).

**TABLE 2:** Fluorescence parameters of HA-pHLIPs in state I (HA-pHLIP in solution at pH8), state II (HA-pHLIP with POPC liposomes in solution at pH8) and state III (Ha-pHLIP with POPC liposomes in solution at pH3), and the mid-points (*pK*) and cooperativities (*n*) of state II to state III transitions are shown.

HA-pHLIP agent	λ_max_ in state I, II and II, nm	State II to state III transition
HA-pHLIP1	352, 350, 339	*pK* = 5.5, *n* = 2.6
HA-Peg12-pHLIP2	352, 350, 340	*pK* = 5.1, *n* = 1.8
HA-Peg12-pHLIP4, v1	347, 343, 337	*pK* = 6.4, *n* = 1.7
HA-Peg12-pHLIP4, v2	348, 343, 337	*pK* = 6.8, *n* = 2.2
2(HA-Peg12)-pHLIP3	351, 351, 337	*pK* = 4.8, *n* = 1.9
2(3xHA-Peg12)-pHLIP3		
2(HA-Peg12)-pHLIP4	347, 344, 337	*pK* = 5.8, *n* = 1.3
2(HA-Peg12)-pHLIP5	347, 346, 340	*pK* = 6.2, *n* = 0.9
2(HA-Peg24)-pHLIP3	351, 350, 337	*pK* = 4.6, *n* = 0.9
2(HA-Peg24)-pHLIP4	347, 347, 336	*pK* = 5.8, *n* = 0.7

It is evident that addition of a polar Peg polymer shifts the *pK* of the transition from state II to state III, which reflects the pHLIP insertion into the membrane, to lower pH values. Thus, the *pK* of HA-Peg12-pHLIP2 is 5.1, while the *pK* of HA-pHLIP1 is 5.5. When two HA-Peg12 units were added (2(HA-Peg12)-pHLIP3) the *pK* is shifted further to 4.8 and even further (*pK* = 4.6) when two HA-Peg24 units are added (2(HA-Peg24)-pHLIP3). The parameters obtained for 2(HA-Peg12)-pHLIP3 and 2(3xHA-Peg12)-pHLIP3 were similar. More hydrophobic versions of the pHLIP peptide, pHLIP4 and pHLIP5, which can be manufactured without difficulty, shifted the insertion *pK* to higher pH values, which are more physiologically relevant. By using revised pHLIP4s, the *pKs* of two versions of the single HA-headed Peg12 construct (HA-Peg12-pHLIP4) were 6.4 and 6.8, and the *pK* of a double HA-headed agent was found to be 5.8. The *pK* of 2(HA-Peg12)-pHLIP5 was shifted even further, to 6.2. The *pK* shift can be understood as arising from stronger hydrophobic interactions of the HA-pHLIP4 and HA-pHLIP5 agents with membranes at normal and high pHs as indicated by the shorter-wavelength fluorescence maximum in state II (343–344 nm for HA-Peg12-pHLIP4 and 2(HA-Peg12)-pHLIP4, and 347 nm for 2(HA-Peg24)-pHLIP4) compared to the 350–351 nm established for the HA-pHLIP1 and HA-pHLIP3 agents. Stronger hydrophobic interactions will place the titratable groups of a pHLIP deeper in the bilayer, where the dielectric is lower and the *pK* is shifted to higher values.

**FIGURE 5:**
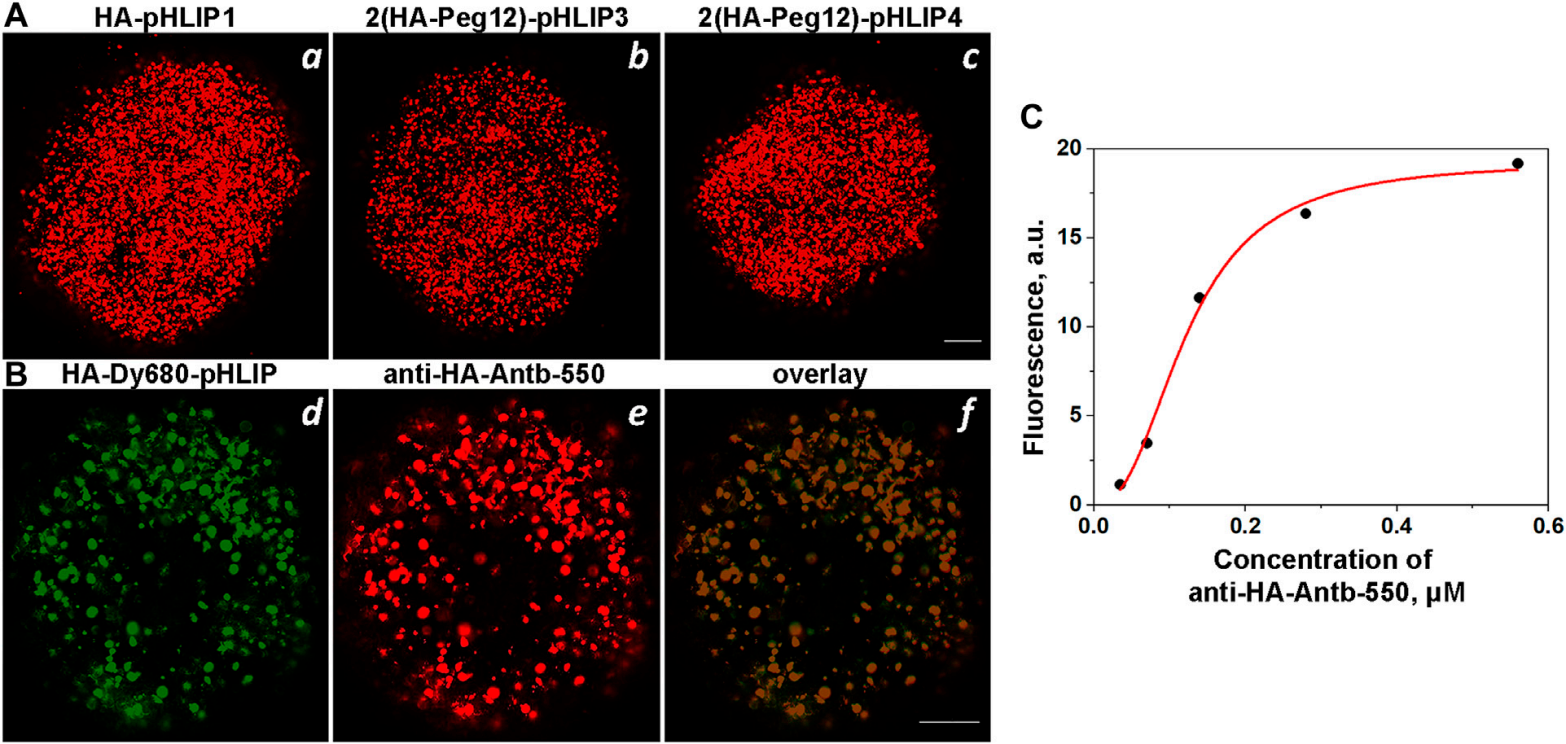
Binging of anti-HA-Antb with HA-pHLIP in tumor spheroids. **(A)**: HeLa tumor cell spheroids were treated with HA-pHLIP1 (a), 2(HA-Peg12)-pHLIP3 (b) or 2(HA-Peg12)-pHLIP4 (c), followed by washing and incubation with anti-HA-Antb-550. Tumor spheroids were then washed and imaged with a confocal microscope. **(B)** HeLa tumor spheroids were treated with HA-Dy680-pHLIP, followed by washing and incubation with anti-HA-Antb-550. Tumor spheroids were then washed and imaged using different channels to visualize fluorescent HA-pHLIP (d), bound anti-HA antibody (e) and their overlay (f) by confocal microscopy using ×10 and ×20 objectives. 100-μm scale bars are shown in all panels. **(C)** Titration curve: HeLa tumor spheroids treated with 1 µM of HA-pHLIP1 followed by washing were then treated with increasing concentrations of anti-HA-Antb-550, the mean fluorescence intensities per area were calculated and are shown vs. the concentration of anti-HA-Antb-550.

HA-pHLIP agents were tested on HeLa and 4T1 tumor spheroids to ensure that the HA epitope is positioned properly at the surfaces of cells so that the anti-HA fluorescent antibody can recognize it. Representative fluorescence images obtained on HeLa tumor spheroids are shown on Figure 5A. In some cases, we used a fluorescent HA-pHLIP agent (HA-Dy680-pHLIP) to establish co-localization with the fluorescent anti-HA antibody (anti-HA-Antb-550), as shown in Figure 5B. We also performed a titration experiment: HeLa tumor spheroids were treated with HA-pHLIP, followed by washing. Next, different concentrations of anti-HA-Antb-550 ranging from 0.035 to 0.56 µM were used to treat the spheroids, followed by washing, imaging and analysis of mean fluorescence per area. The fluorescence signal reflects the binding of anti-HA-Ant550. The signal increases with the increase in antibody concentration until saturation is achieved (Figure 5C), clearly indicating specific binding of anti-HA antibody with HA epitopes.

**FIGURE 6:**
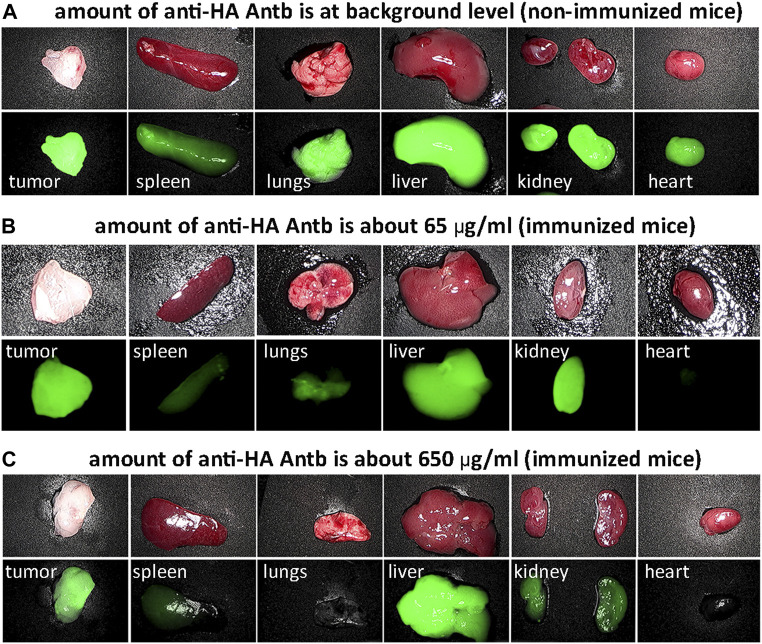
HA-pHLIP tumor targeting and biodistribution. Photos and NIR fluorescent images of major organs collected 24-h after administration of HA-ICG-pHLIP to mice bearing 4T1 flank tumors: non-immunized mice containing no anti-HA antibodies **(A)** and immunized mice with low **(B)** and high titer of anti-HA antibodies **(C)**.

We investigated the biodistribution of fluorescent HA-pHLIP agents. Fluorescent HA-ICG-pHLIP was administered as a single IP injection to non-immunized mice and immunized mice with different anti-HA antibody titers (Figure 6). All mice had 4T1 tumors. We employed different schemes of immunization to establish different amounts of anti-HA antibodies in the blood of the animals. About 65 μg/ml of anti-HA-Antb was established in one of the immunized groups and about 650 μg/ml in another immunized group. Significant tumor targeting was achieved in all groups. At the same time, the fluorescence signals in the organs were observed with the increase of the amount of anti-HA antibodies in the blood. The signal in the spleen, lungs and heart decreased and was essentially undetectable in immunized mice with a high titer of anti-HA antibodies. The signal in the kidney dropped as well, while a signal in the liver was observed in all groups, indicating hepatic clearance. A more detailed biodistribution study was performed with ICG-2(HA-Peg12)-pHLIP4, which was given as a single injection into mice immunized with KLH-HA/AddaVax (Supplementary Figure S2). After immunization, 4T1 tumors were grown and mice received a single IP injection of ICG-2(HA-Peg12)-pHLIP4 (40 μM 400 μl). Mice were euthanized at 24-, 8- and 72-h, tumor and major organs were collected and imaged. The signal levels in all organs at different time points are shown on Supplementary Figures S2A–C and kinetics of fluorescence signal changes are shown in Supplementary Figure S2D. The signal in all organs decayed, indicating clearance of the agent, while the signal in the tumors was persistent. It is important to note that the signal in spleen, lungs, kidney and heart of immunized mice was much lower compared to the signal in these organs of non-immunized mice. It is expected that HA-pHLIP binds anti-HA antibodies in the blood and potentially travels as an HA-pHLIP antibody complex and therefore has limited accessibility to major organs, resulting in a lowered signal in the organs of immunized mice with a high amount of anti-HA antibody. We checked the level of anti-HA antibody before injection of HA-pHLIPs and 24-, 48-, and 72-h later, when animals were euthanized. The level of anti-HA antibodies in the serum dropped 4.5 fold from 65 μg/ml to 14.6 μg/ml 24 h after injection of ICG-2(HA-Peg12)-pHLIP4, indicating binding of HA-pHLIP with anti-HA antibodies (Supplementary Figure S2E). The level of unbound anti-HA antibodies slowly increased in the blood over the next few days.

**FIGURE 7:**
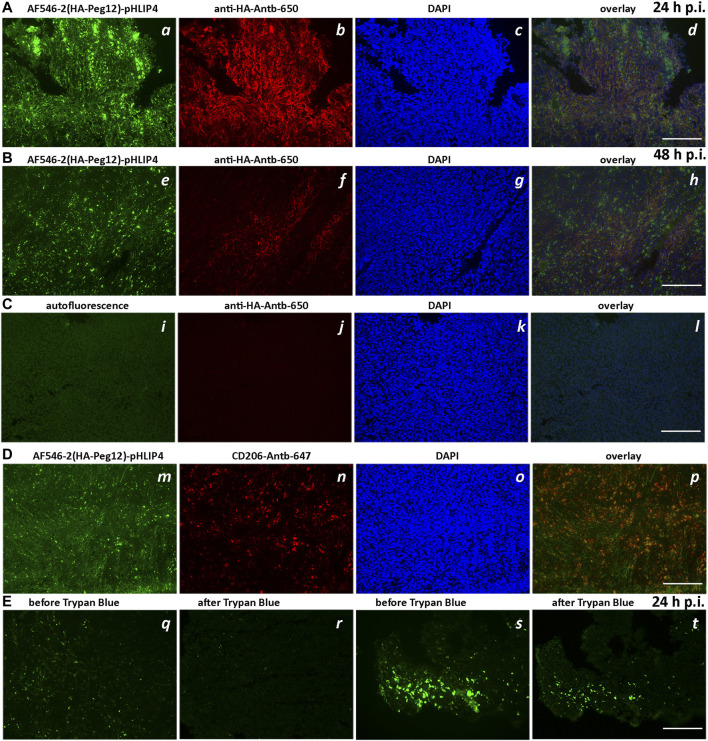
Extracellular exposure of HA epitope in tumors. Fluorescence images of tumor sections obtained from mice injected with AF546-2(HA-Peg12)-pHLIP4 (40 μM 400 μl) **(A,B,D,E)** and control mice, which did not receive injection of HA-pHLIP agent **(C)**. The fluorescence images of AF546-2(HA-Peg12)-pHLIP4 (a,e,m), sections stained with anti-HA-Antb-650 (b,f,j), or CD206-Antb-647 (n) and DAPI (c,g,k,o), and overlay images (d,h,l,p) (image d is an overlay of 3 images a, b, and c; image h is an overlay of 3 images e, f, and g; and image l is an overlay of 3 images i, j, k, and image p is an overlay of 2 images m and n) are shown for tumors resected 24-h **(A)** and 48-h **(B)** after a single IP injection of AF546-2(HA-Peg12)-pHLIP4. **(C)** represents images of tumor sections obtained from control mice [no injection of HA-pHLIP agents (i)], while sections were stained with anti-HA-Antb-650 (j) and DAPI (k). In **(E)**, two sets of images from unfixed tumor sections obtained from the mice injected with AF546-2(HA-Peg12)-pHLIP4 are shown before (q,s) and after (r,t) addition of solution of Trypan Blue to the sections. The images were acquired using a fluorescence inverted microscope. 200-μm scale bars are shown.

In the next set of experiments, we evaluated the ability of pHLIP to position and expose HA epitopes to the extracellular surface for anti-HA antibody recognition within tumors. Mice bearing 4T1 tumors in their right flanks received single IP injections of AF546-2(HA-Peg12)-pHLIP4 (40 μM 400 μl). At 24-, 48- and 72-h post injection, the mice were euthanized, and tumors were resected, frozen and sectioned. Sections were stained with fluorescent anti-HA, or CD206 antibodies and DAPI, or H&E. At 24-h after administration of AF546-2(HA-Peg12)-pHLIP4, the fluorescence signal of HA-pHLIP and anti-HA antibody were observed within tumor sections (Figure 7A). The majority of the HA-pHLIP fluorescence is co-localized with anti-HA antibody fluorescence (Figures 7A, d). At 48-h p.i., despite the fact that HA-pHLIP fluorescence was present, the amount of anti-HA antibody recognizing HA-pHLIP was less (Figure 7B). Sections from the control mouse with no injection of HA-pHLIP are shown on Figure 7C. At 72-h, no anti-HA antibody was bound to cells (data not shown). “Hot spots” for HA-pHLIP fluorescence signal were detected. These “hot spots” were mostly not stained by anti-HA antibody, potentially indicating that HA-pHLIP was internalized by some cells and was therefore not accessible to anti-HA antibody binding. Further investigation revealed that the “hot spots” of HA-pHLIP fluorescence were co-localized with CD206 antibody staining of tumor-associated macrophages (TAMs) (Figure 7D). We also employed the Trypan Blue quenching assay, which allows to distinguish the extracellular signal from the intracellular, since Trypan Blue is a cell-impermeable quencher of fluorophores emitting in the range of 500–600 nm. Tumor sections, either containing or not containing contain “hot” AF546-2(HA-Peg12)-pHLIP4 fluorescent spots, were imaged before and after Trypan Blue addition (Figure 7E). Most of the HA-pHLIP fluorescence was quenched by Trypan Blue indicating extracellular localization of HA-AF546. However, some “hot” spots remained fluorescent, indicating internalization of HA-AF546 by cells (mostly TAMs).

**FIGURE 8:**
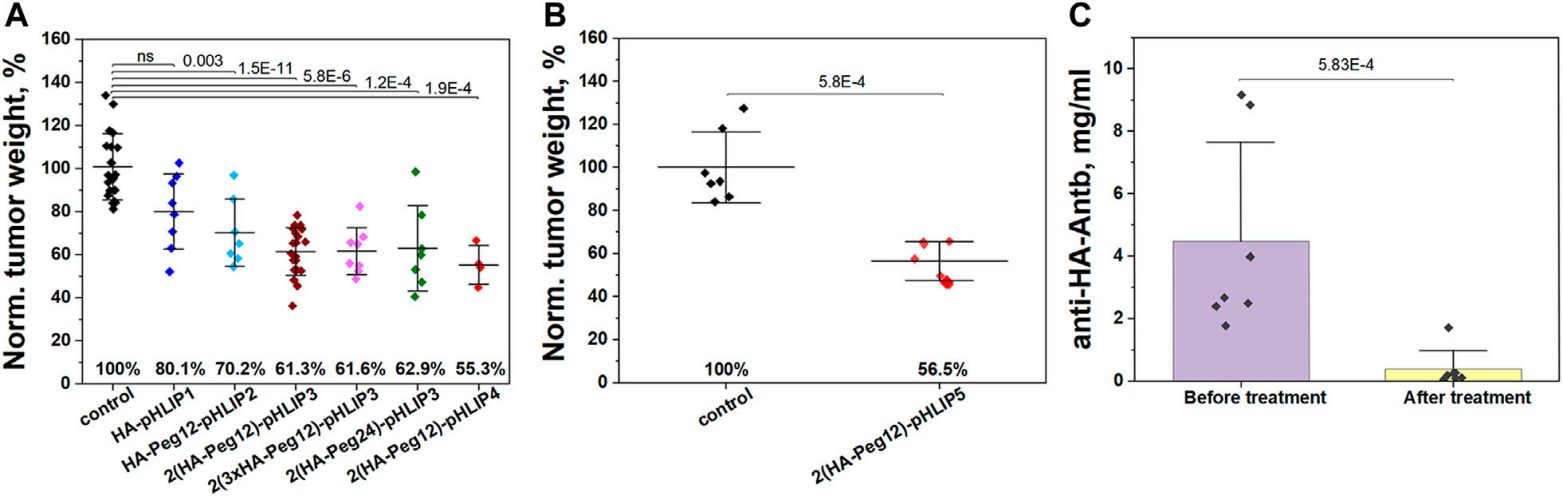
Therapeutic efficacy of HA-pHLIP. **(A)** Normalized weight of murine breast 4T1 tumors collected from mice immunized with KLH-HA/CFA and KLH-HA/IFA and treated with HA-pHLIP agents is shown. **(B)** Normalized weight of murine melanoma B16F10 tumors collected from mice immunized with 2 injections of KLH-HA/AddaVax and treated with 2(HA-Peg12)-pHLIP5 is shown. The mean weight of tumors in the control group was taken as 100%. At the 0.05 level the distributions of tumor weight in the control (untreated) group and HA-pHLIP (treated) groups are statistically different as established by the Kolmogorov-Smirnov two-tailed non-parametric test (except for the HA-pHLIP1 agent in panel A). **(C)** The amount of anti-HA antibody in serum obtained from mice (bearing B16F10 tumors) before the treatment was initiated and after treatment with 2(HA-Peg12)-pHLIP5 was completed and animals were euthanized. Statistical significance was established by the Kolmogorov-Smirnov two-tailed non-parametric test.

We evaluated the therapeutic efficacy of the HA-pHLIP agents on immunized mice bearing 4T1 flank tumors. Mice were immunized by injections of KLH-HA/CFA and boosts of KLH-HA/IFA. Blood was collected randomly from several mice to confirm high titers of anti-HA antibody prior to the injection of cancer cells. Cancer cells were inoculated on day 1 and treatment was initiated on day 3. IP injections of various HA-pHLIP agents were performed every second day and continued until all mice were euthanized, tumors were collected and weighed. The treatment results are presented in Figure 8A. In another control experiment, the selected HA-pHLIPs were injected into non-immunized mice with no effect on tumor growth (data not shown). We also performed multiple injections of HA-pHLIP to establish if anti-HA antibodies could be developed, and found no detectable antibodies. Statistically significant therapeutic effects were established between control and treated groups except for HA-pHLIP1, probably indicating that more space is needed between the epitope and pHLIP for better exposure of the epitope to antibody binding. However, no difference was observed between agents with Peg12 and Peg24 spacers between the epitope and pHLIP. Also, no difference was noted between the performance of the HA-pHLIP agent with a single HA epitope and the agent with 3 repeats of the HA epitope (3xHA), most probably because the affinity to the anti-HA antibody was not altered. The more hydrophobic agent (pHLIP4), with its *pK* shifted toward higher pH values, exhibited a slightly better therapeutic performance, however statistical significance was not achieved. In another experiment, we treated mice immunized with 2 injections of KLH-HA with AddaVax adjuvant. Melanoma B16F10 cancer cells lacking MHC-I presentation were inoculated in right flank of mice after immunization was completed. Tumors were grown until they reached volumes of about 100 mm^3^, which was designated as Day 1. Three consecutive IP injections of 2(HA-Peg12)-pHLIP5 agent (40 µM 400 µl per injection) were given (one injection per day) on days 1, 2 and 3. The tumor in the control group reached a volume of about 1,500 mm^3^ on day 7 and all animals in this group were euthanized. Normalized tumor weights from the control (non-treated) and treated with 2(HA-Peg12)-pHLIP5 groups are shown on Figure 8B. We also established the amount of anti-HA antibodies in all mice in the treated group before the treatment was initiated and after the treatment was completed and animals were euthanized (Figure 8C). The amount of anti-HA antibody dropped significantly (close to background level) after 3 injections of HA-pHLIP and the level of antibodies was not restored by day 7. The drop of antibodies is therefore likely to be limiting the observed therapeutic effect. Just two injections of the HA-pHLIP agent were sufficient to capture practically all of the anti-HA antibodies in the blood. Successful continuation of the treatment might require additional boosts to restore the amount of anti-HA antibodies and keep them at high levels. Importantly, none of animals in the treated group lost weight during the treatment, and all resected organs appeared normal.

We also tested several different adjuvants since the CFA adjuvant cannot be translated to the clinic. The amount of anti-HA antibodies after 2 injections of KLH-HA/CFA and KLH-HA/IFA was in the range of 200–400 μg/ml. Similar antibody concentrations resulted from using the IFA adjuvant. A lower level (50–100 μg/ml) resulted from 2 injections using AdjuPhos, which is an aluminum phosphate wet gel suspension used as an adjuvant in humans for many years, and which predominantly activates the Th2 helper cell response leading to the production of antibodies. Two injections with the AddaVax adjuvant, a squalene-based oil-in-water nano-emulsion with a formulation like the FDA-approved novel MF59^®^ adjuvant used with flu vaccines, produced the highest amount of anti-HA antibodies in the range of 1–3 mg/ml. AddaVax activates both the Th1 and Th2 helper cell immune responses for training of both T- and NK-cells and the production of antibodies. We also tested commercially available Flu vaccines approved for canines (Vanguard CIV H3N2) and for humans (Flucelvax^®^ Quadrivalent) since the HA sequence is from the hemagglutinin protein of the H3N2 influenza virus. Neither vaccine induced production of anti-HA antibodies in mice, indicating that dog and human vaccines are not suitable to initiate anti-HA immunity. However, humans who have had a prior infection with the H3N2 flu virus might develop anti-HA immunity in the course of the disease, since HA is a highly immunogenic epitope. We tested plasma samples from 20 healthy humans and established the level of anti-HA antibodies. The histogram presented in Supplementary Figure S3 indicates that one person had < 10 μg/ml of anti-HA antibodies in the blood. Nine, four and two people had levels in the ranges 10–20, 20–30 and 30–40 μg/ml, respectively. Four people had levels of anti-HA antibodies higher than 40 μg/ml.

## 4 Discussion

Immune check point inhibitors (ICIs), which promote the cytotoxic action of T cells, lead to significant therapeutic effects in some cancer patients. However, the response rates are not high, ranging from 20% to 40% (Sharma et al., 2021). Preclinical and clinical evidence suggests that ICIs/T cell based therapies do not work in “cold” non-inflamed tumors with impaired MHC-I (major histocompatibility complex class I) presentation and low tumor mutational burden (TMB) (Volz et al., 2019; Bai et al., 2020; Cornel et al., 2020; Dhatchinamoorthy et al., 2021; Hodi et al., 2021; Liu and Sun, 2021). Therefore, other immuno oncology therapeutic approaches are under development, including the delivery of antigens (and epitopes) aimed at triggering immunological responses by activating of NK-cells. Since pHLIP peptides target tumor cells and insert across their membranes, and since the insertion leaves one end of the peptide exposed at the cell surface, we have investigated the idea of attaching an epitope to the extracellular end of pHLIP for targeted extracellular delivery of epitopes to tumors as a way of inducing an immune response to the tumor cells.

We tested pHLIP-mediated targeted delivery of DNP and α-Gal epitopes to cancer cells in tumor spheroids and in mouse tumor models. Both epitopes were properly targeted and positioned extracellularly and were exposed to corresponding anti-DNP and anti-Gal antibodies. No therapeutic effect of DNP-pHLIP was observed, while treatment with Gal-pHLIP induced regression of MHC-I negative melanoma B16F10 tumors. Both approaches, if transferred to the clinic, would rely on the presence of endogenous anti-Gal and anti-DNP antibodies in humans, while in the mouse experiments an immunization is necessary. The repertoire of useful epitopes could be significantly expanded if targeted delivery of an antigen/epitope were coupled with immunization to develop corresponding anti-epitope antibodies, prompting us to investigate this direction. Currently, vaccination for cancer prevention and treatment is based on the need to identify neo-antigens at the surfaces of cancer cells, and then to perform immunization to activate T- and NK-cells and thereby to develop anti-antigen antibodies (Finn, 2018; Crews et al., 2021). An alternative approach is to perform an immunization and induce production of a high titer of antibodies against an exogenous antigen, not present in the human body, and then to position this antigen on cancer cells, which will be recognized by newly produced antibodies to induce ADCC, ADCP and CDC.

As an exogeneous immunogenic antigen we selected the 9 amino acid HA peptide that corresponds to amino acids 98-106 of the human influenza virus hemagglutinin (Wilson et al., 1984). The HA peptide has been extensively used as a general epitope tag in expression vectors in HA-tagged fusion proteins (Field et al., 1988). We have shown that immunity is readily developed for the HA peptide in mice, as shown by the high blood levels of anti-HA antibodies after immunization with KLH-HA in the presence of adjuvants. A high immunogenicity for such a short peptide epitope in humans might be explained by the absence of the HA peptide sequence in the genome. Typically, immunogenicity for carbohydrate epitopes or for similar sized peptide epitopes is low. We would like to note that KLH is used in humans and that translation of KLH-HA paired with an FDA-approved novel MF59^®^ adjuvant (in some experiments we used Addavax, a squalene-based oil-in-water nano-emulsion with a formulation similar to MF59^®^) is expected to be straightforward. We explored the possibility of using human or dog flu vaccines against the H3N2 influenza virus. No anti-HA antibodies were developed in mice. It is not surprising that the existing Flu vaccines do not give immunity to the HA peptide, since the 98–106 sequence of HA peptide is located in the trimer interface of the hemagglutinin and is not accessible to antibodies (Wilson et al., 1984), and vaccines mostly are developed for the parts of viral proteins that are readily accessible (Rajao and Perez, 2018). At the same time, individuals who have contracted the H3N2 flu virus might develop anti-HA immunity, since the peptide could be exposed and recognized as foreign during the natural virus-elimination process. We tested plasma samples from 20 healthy donors and found variable amounts of anti-HA antibodies. Individuals who recently contracted the H3N2 virus might have a high titer of anti-HA antibodies, while others with a low titer could be immunized with KLH-HA/MF59^®^ to achieve a higher titer. Flu vaccination is not expected to interfere with the process. We also note that after treatment the HA peptide will likely be destroyed and immunoreactivity will be suppressed, since the DVPD sequence from the HA peptide is cleaved by the Caspase-3 and Caspase-7 overexpressed in apoptotic cells (Schembri et al., 2007). Thus, use of the HA epitope seemed to be an attractive choice of epitope from several standpoints, motivating our studies with pHLIP peptides.

In addition to testing HA simply attached to the end of a pHLIP, we tried to enhance the overall binding affinity of HA-pHLIP to anti-HA antibodies using double headed HA-pHLIP agents, where HA epitopes were linked to a single pHLIP peptide *via* Peg12 or Peg24 polymers. However, the attachment of these large, polar entities shifted the *pKs* of insertion of the HA-pHLIP agents to lower pH values that are less relevant to the values of pH at the surfaces of cancer cells (Anderson et al., 2016; Wei et al., 2019). Tuning of pHLIP properties and introducing more hydrophobic sequences allowed restoration of suitable *pKs* of insertion. In experiments using tumor spheroids, all agents targeted acidic cells and positioned HA epitopes in the extracellular space accessible to anti-HA antibodies. Fluorescent versions of the agents were used to demonstrate excellent tumor targeting and clearance of the agent from most organs. The biodistribution observed in non-immunized and immunized mice with different titers of anti-HA antibodies was different: the agent was widely distributed in the organs of non-immunized mice, but not found in the major organs of immunized mice with high amounts of anti-HA antibodies, except for the liver, the organ of clearance. Also, the level of anti-HA antibody in the blood dropped by 77.5% 24-h after HA-pHLIP administration. These data indicate that agents can bind to anti-HA antibodies in the blood of immunized mice and travel as antibody complexes to target tumors.

The HA antigens should be positioned and remain at cell surfaces to induce immune suppression of tumors *via* ADCC, ADCP or CDC. For 24–48 h the HA epitope is exposed to the extracellular space, while later it is internalized by cells. Thus, the time window for therapeutic action for a day after tumor targeting is achieved. Some of the agent is taken up by macrophages and may potentially be internalized even faster. Our main finding is that treatments of imunized mice, bearing 4T1 triple negative or B16F10 MHC-I negative melanoma flank tumors, with a double-headed HA-pHLIP resulted in a 55% reduction of tumor growth. We demonstrated that practically all of the anti-HA antibodies in the blood were captured in complexes after 3 injections of HA-pHLIP. Such a capture depletes the effective antibodies and may well limit therapeutic efficacy in the mouse tumor models. Additional boosts might be used to restore the level of free anti-HA antibodies, however the tumors in our models were developing too fast to effectively employ additional boosts in the course of the treatment. Additional experiments might be performed on slow-growing tumor models, when multiple boosts could be done to keep the amount of anti-HA antibodies at a high level, potentially enabling a more significant therapeutic efficacy.

Immunization coupled with epitope targeting might open an opportunity for the treatment of tumors with a low number of neo-antigens (or low TMB) and absence or low MHC-I presentation. These tumors are problematic for treatments based on T-cell action and for the development of anticancer therapeutic vaccines. In the work reported here, prior immunization ensures a high titer of anti-HA antibodies, and the pHLIP-targeted delivery of HA epitopes to position them on the surfaces of cancer cells results in antitumor activity. Of course, the approach might be combined with any therapies that promote the presence and activation of NK-cells within the tumor microenvionment.

The idea to treat tumors by marking cancer cells with pHLIP-targeted exogeneous antigens opens an opportunity either to exploit endogeneous antibodies present in the organism or to employ new ones by immunization against the desired antigens.

## Data Availability

The raw data supporting the conclusion of this article will be made available by the authors, without undue reservation.
