# Supplementary figures and images for: Tumor treatment by pHLIP-targeted antigen delivery
Source: Front Bioeng Biotechnol. 2023 Jan 6;10:1082290. doi: 10.3389/fbioe.2022.1082290 (PMC9853002; doi:10.3389/fbioe.2022.1082290)

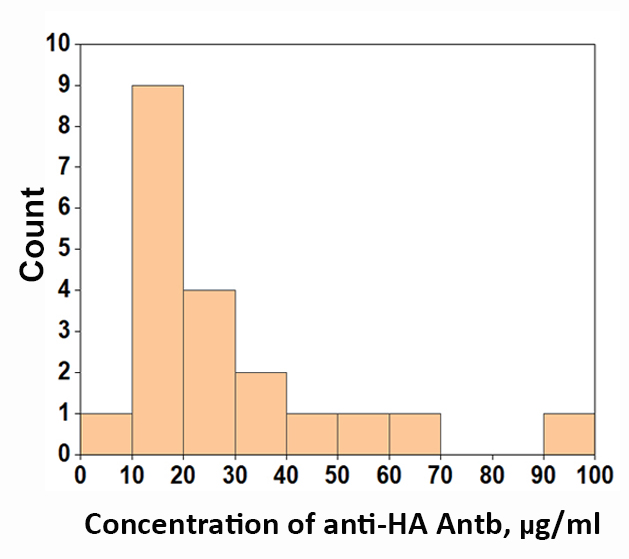

Supplement: Supplementary file 1 [file Image3.jpg]

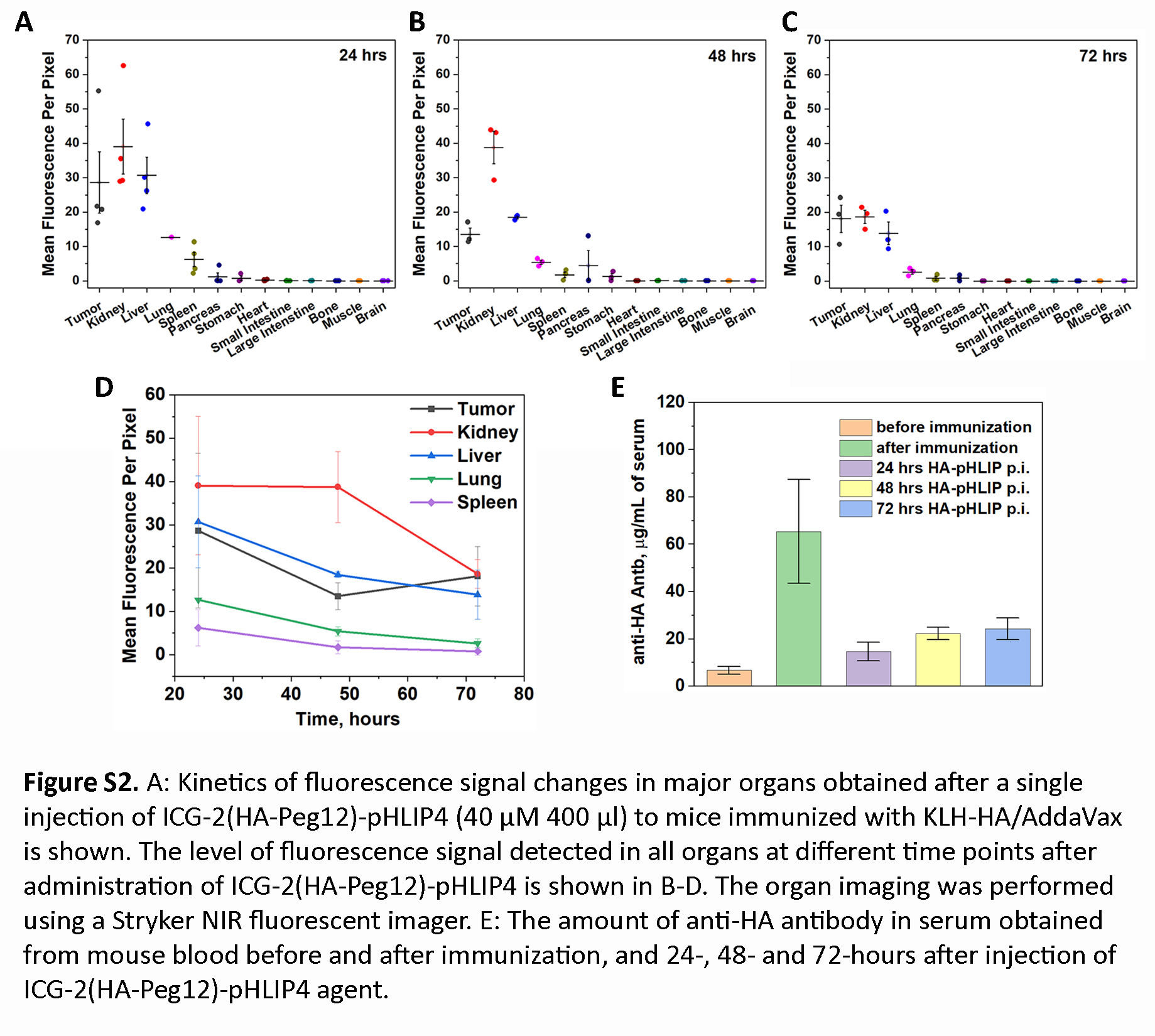

Supplement: Supplementary file 2 [file Image2.jpg]

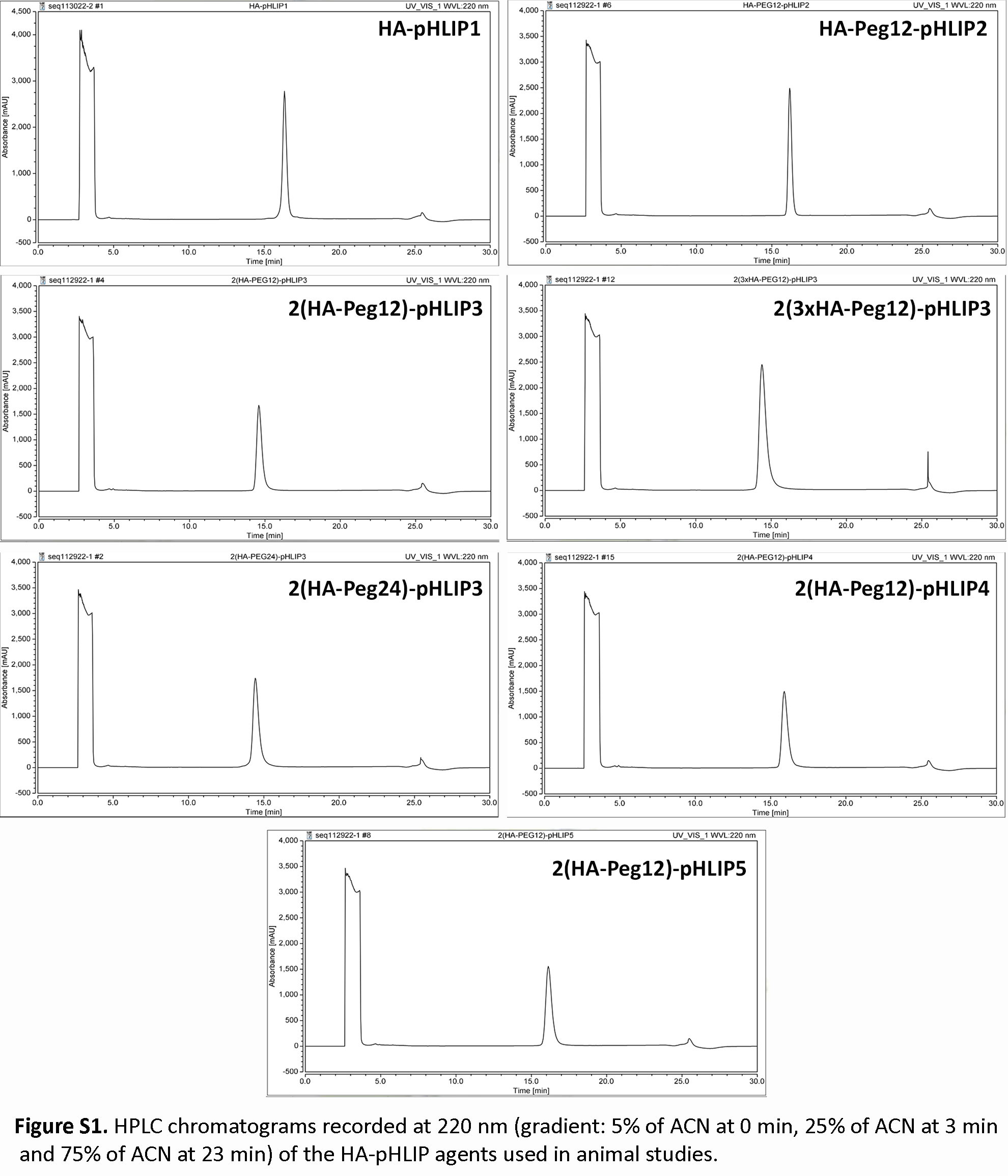

Supplement: Supplementary file 3 [file Image1.jpg]
